# Awareness and compliance with pharmacovigilance requirements amongst UK oncology healthcare professionals

**DOI:** 10.3332/ecancer.2018.809

**Published:** 2018-02-08

**Authors:** Rebecca J Thorne, Rosanne J Bruggink, Stephen J Kelly, Sarah JL Payne, Simon J Purcell, David A Montgomery

**Affiliations:** 1 Aston University, Aston Expressway, Birmingham B4 7ET, UK; 2 The University of Sussex, Falmer, Brighton BN1 9RH, UK; 3 Pfizer Ltd, Walton Oaks, Dorking Road, Surrey KT20 7NS, UK

**Keywords:** pharmacovigilance, pharmacists, oncology nursing, surveys and questionnaires, marketing, oncologists

## Abstract

Since 2013, once a medicine receives marketing authorisation in the European Union, it is labelled with an inverted black triangle indicating all adverse reactions should be reported. Our aim was to explore understanding of the black triangle and compliance with adverse event (AE) reporting requirements by UK oncology healthcare professionals (HCPs). A questionnaire was electronically distributed to oncology pharmacists (P) via the British Oncology Pharmacy Association, to oncologists (O) through the Association of Cancer Physicians and also to nurses (N) via the UK Oncology Nursing Society. Overall, 125 (42 O, 61 P, 22 N) clinicians participated. The purpose of the black triangle was unknown by 26% (55% O, 5% P, 28% N) and 54% did not alter their AE reporting in the presence of a black triangle. Once the black triangle was removed, only 38% were aware which AEs should be reported, 46% did not report all serious AEs for established medicines, including life-threatening or disabling AEs. Reasons for non-reporting were decision making on what to report (45%); time consumed by reporting (41%); AEs perceived as not serious enough (35%) and follow-up process (23%). Understanding of the pharmacovigilance framework among respondent groups was variable. Across all groups, AEs appear substantially under-reported. Reasons identified in the study include the time consuming nature of AE reporting and a lack of understanding around the black triangle and AE reporting process. There is a need to further support HCP education on AE reporting coupled with a review of the current reporting process to ensure maximal engagement.

## Introduction

Pharmacovigilance, the act of detecting, assessing, understanding and preventing adverse effects or drug related complications, is critically important to understand the complete safety profile of a medicine. While the adverse event (AE) profile may be generally well understood at launch, understanding of rare or very rare AEs or those AEs with long latency will be limited at the time of license. These rare events can often be serious, and so spotting the association early is crucial to minimise the risk of harm to patients.

In 1965, in the wake of spotting in the post marketing setting the association between birth defects in children whose mothers took the anti-emetic thalidomide during pregnancy, the Medicines and Healthcare product Regulatory Agency (MHRA) set up the Yellow Card Scheme, allowing a national adverse drug reaction (ADR) reporting system [1]. Expansion of the scheme means that all healthcare professionals (HCPs) and members of the public are now able to report ADRs to the MHRA via the Yellow Card Website, phone, post or app [2]. Despite regulatory recommendations, AEs are recognised as under-reported; it is thought that only 10% of serious ADRs and 2–4% of non-serious ADRs are reported in the UK [1].

To optimise signal detection and minimise the ‘noise’ of AE reporting that can cloud true associations, not all AEs need to be reported. For established medicines, only serious or unexpected AEs need to be reported [2, 3]. But enhanced reporting is required for new active substances or biosimilars, new combinations of medicines or active substances, a new route of administration, a new drug delivery system or an established drug to be used in a new patient population [4] and medicines subjected to enhanced reporting must display an inverted black triangle on package leaflets, HCP information, advertising and the SmPC. The black triangle scheme has been running in the UK for a number of years but in 2013, it was extended across the European Union (EU).

There is no national ADR curriculum for pharmacy and medical schools; however, the Yellow Card Scheme is included in most undergraduate programmes, although an expectation of Good Clinical Practice ADR training is not a part of hospital annual training [5]. Five Yellow Card Centres, run on behalf of the MHRA, and based in Newcastle, Liverpool, Cardiff, Birmingham and Edinburgh, aim to improve drug safety by raising awareness of ADRs, enhancing the number of spontaneous ADR reports, improving education on ADRs to undergraduates and clinicians, and promoting research that facilitates better understanding of the causes, effects and avoidance of ADRs [6, 7].

In the UK, it is well recognised that HCPs under-report AEs mainly due to time constraints, uncertainty over responsibility for reporting and lack of knowledge [8–12]. In 2000, it was observed that hospital pharmacists who received ADR training were more likely to report ADRs; however, factors such as time, confidence and concerns for patient confidentiality all reduced reporting. Training and meetings were suggested methods of improving reporting [10]. Green and colleagues reported a reasonable knowledge of the Yellow Card by hospital pharmacists: most knew that all reactions should be reported for newly marketed drugs and only serious reactions for established products [9]. In contrast, a study of medical practitioners found that hospital doctors were less aware than doctors in general practice of the criteria for reporting ADRs and less than half of all doctors knew the precise meaning of the black triangle [8].

## Aim

As oncology products become more targeted, nuanced and specific to smaller populations, AE reporting systems could provide significant information in the real-world use and application of these products. No study has thus far specifically looked at awareness and compliance of pharmacovigilance requirements within the oncology profession, and therefore, we aimed to explore under-reporting of AEs, barriers for AE reporting and clinician awareness of the black triangle in the real world amongst UK oncology HCPs. We expect engagement with AE reporting programmes will be poor and that the significance and implications of the black triangle are not recognised by oncology HCPs.

## Methods

Using an online survey tool, a questionnaire consisting of 12 questions split in to 3 sections (AE reporting, black triangle and prescribing information (PI)) was devised to measure engagement with AE reporting, barriers to AE reporting and whether the significance of the black triangle is recognised amongst oncology HCPs. A full list of the survey questions is given in Appendix 1. This paper discusses the black triangle and AE aspects of the survey; therefore, the final three questions on PI were omitted from results and discussion. Responses to the questions were not mandatory, those who selected ‘never reported an AE’ in question 2 were not asked about how they reported AEs or if they report all serious AEs and automatically skipped to question 5.

The British Oncology Pharmacy Association (BOPA), Association of Cancer Physicians (ACP) and UK Oncology Nursing Society (UKONS), each distributed a different survey link to their members by email. For the survey to UKONS, the final three questions on PI were omitted as it was felt these were not relevant to non-prescribing nursing professionals. Data collection occurred over 12 weeks with access to the survey for 12, 10 and 4 weeks for BOPA, ACP and UKONs, respectively. Participants were thanked on completion of the survey for their responses and given links for more information on Yellow Card reporting and the black triangle.

## Results

We received one hundred and twenty-five responses of which 87% were fully complete. Responses totalled 72 from BOPA, 42 from ACP and 11 from UKONS and were from 42 Oncologists (O), 22 nurses (N) and 61 pharmacists (P). Surveys received via BOPA completed by nurses totalled 11 and these were analysed with the results from UKONS.

Of note, 17% (19% O, 12% P, 27% N) answered that they had never reported an AE; 41% (57% O, 34% P, 27% N) reported one per year; 27% (14% O, 41% P, 14% N) reported 2–4 per year; 8% (2% O, 8% P, 18% N) reported 5–9 per year; and 7% (7% O, 5% P, 14% N) reported more than 10 per year. For those who have previously reported an AE, when asked if they reported all serious AEs for established medicines oncologists were less likely to report with 62% answering no versus 43% of pharmacists and 25% of nurses, giving an average of just over half reporting consistently.

**Figure 1.**
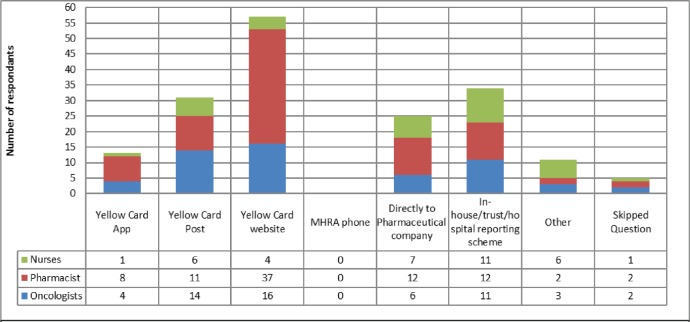
Methods used by oncology HCPs to report adverse events. 104 respondents, who had previously reported an adverse event, were able to select multiple methods of reporting. The Yellow Card Website is the most used route followed by in-house reporting and postal response Yellow Cards.

**Figure 2.**
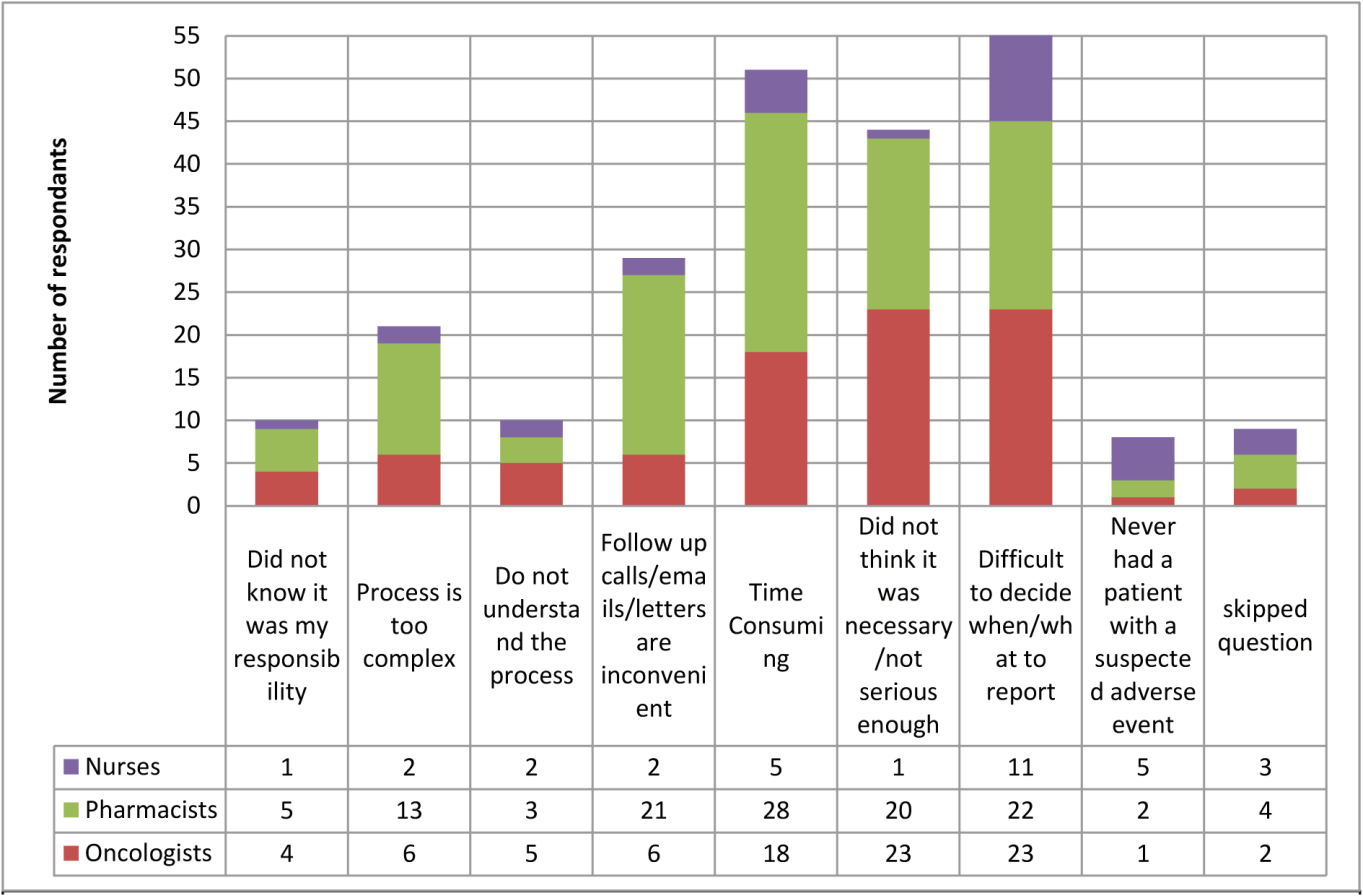
Reasons oncology HCPs do not reporting all adverse events. 104 respondents, who had previously reported an adverse event, were able to select multiple reasons for not reporting. Difficulty in deciding what to report was the most likely reason for notsubmitting an adverse event followed by the time consuming process.

When asked about reporting routes (Figure 1), it is clear that the Yellow Card Website was by far the most common method to report AEs with 62 respondents using this method. In-house reporting schemes and direct to company were the next most common, it is unclear whether in-house reporting schemes subsequently see reports made to the MHRA or to companies. This would be an important area to conduct follow up work, given the relatively common use of this approach. Limitations that result in AEs not being reported are shown in Figure 2, the main reason for not reporting all AEs was difficulty in deciding when and what should be reported, with 45% of respondents selecting this answer.

Responses indicated that 65% knew the correct definition of the black triangle; however, there were substantial variations between specialties, with only 38% of oncologists knowing the correct answer compared to 87% of pharmacists. Nurses responded correctly in 55% of cases. Overall, 17% (38% O, 0% P, 23% N) selected ‘I don’t know’ and 9% (17% O, 5% P, 5% N) selected an incorrect answer suggesting pharmacists are more familiar with the black triangle and its meaning than nurses and oncologists. When HCPs were asked what should be reported following the removal of the black triangle, 15% (5% O, 20% P, 23% N) answered that all AEs should be reported; 54% (38% O, 70% P, 41% N) answered serious AEs only; 58% (72% O, 57% P, 32% N) answered unexpected AEs only; 2% selected no AEs needed to be reported if a medicine does not have a black triangle. 38% correctly answered that both serious and unexpected AEs should be reported, with 54% pharmacists answering correctly versus 26% oncologists and 18% nurses, highlighting pharmacists’ relatively better knowledge on the subject. Over one-third (36%) did not know that the black triangle is removed once the safety of the drug is established. The presence/absence of a black triangle did not alter AE reporting habits of 54% (71 % O, 39 % P, 64 % N).

## Discussion

In the UK, AEs are consistently under-reported [8, 9]. The extent of under-reporting is likely to be underestimated due to different studies using various methods to calculate under-reporting. Hazell and colleagues reported that across eight studies investigating ADR reporting in hospitals worldwide, the under-reporting rate ranged from 59% to 100% [13]. In our survey almost half of those that had previously reported an AE did not believe they report all serious AEs and only 7% reported more than ten a year. This suggests a dramatic scale of under-reporting in a specialty where AEs are routine. Oncologists were more likely not to report serious AEs. The most frequent barriers to reporting were deciding if it is necessary or serious enough for the AE to be reported and time constraints. These findings are consistent with previous studies [8–11].

Vallano and colleagues described lack of knowledge on pharmacovigilance systems within hospitals as an obstacle for reporting; however, only 8% in the current study reported not understanding the process. Of oncology pharmacists, 38% did not report all AEs due to difficulty deciding whether or not an ADR had occurred [11]. This is consistent with a review in 1996 about the attitude of the hospital pharmacist towards ADR reporting which found that 32% were discouraged from reporting ADRs for the same reason [9]. This suggests that although the process of reporting is understood, finding time and deciphering what to report remain a challenge.

The black triangle has been an important part of UK pharmacovigilance further indicated by its extension to an EU wide scheme in 2013. It was previously suggested that hospital pharmacists are more likely to report serious and rare ADRs for newly marketed drugs and 34% suggested further training would likely increase ADR reporting [10]. Although the study by Sweis and colleagues was not specific to oncology pharmacists, increased reporting of ADRs in newly marketed drugs could be related to 87% of pharmacists knowing the definition of the black triangle compared with 43% of registrars, consultants and nurses in this study. Our findings suggest that more training is required for medical professionals on the pharmacovigilance framework in the UK.

## Conclusion

The consistent outcome from the study across a variety of oncology HCPs was under-reporting of drug related AEs and lack of black triangle knowledge. Pharmacists have a reasonable understanding of the definition of the black triangle (although not complete). Nurses and oncologists had a poor understanding. Overall, there is a need to further support HCP education around AE reporting and the black triangle to ensure confidence in making reports and that the importance and ultimate benefit to patients of robust AE reporting are clear.

## Study limitations

There were some limitations to this research:

- Our method of distribution reached a wide group, but being an electronic distribution list our actual denominator for the survey respondents is not known, meaning we are not clear on the response rate.
- Respondents to the survey were self-selected; the findings in the wider population could be better or worse than we have seen.
- We avoided free text boxes. The correct answers could have been guessed by respondents (or even looked up), meaning our findings could over-estimate the knowledge of the respondents

## Further research

As a follow-up, we suggest contacting schools of medicine and pharmacy to analyse how training on ADR reporting is delivered with the aim of aligning this with the Yellow Card Centres model of advice and training. A review of the current reporting process and a survey of changes HCPs would make to improve the current process would be beneficial to increase engagement with reporting.

Following on from this research, to assist in educating HCPs, we have developed an educational video on the Black Triangle and ADRs (Video 1).

## Figures and Tables

**Video 1.**
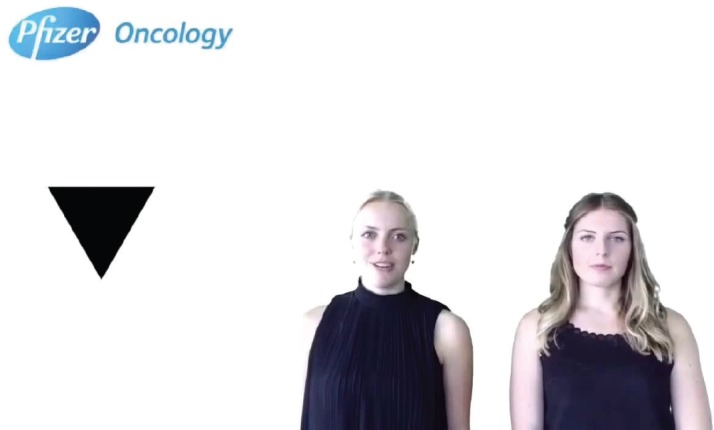
The Black Triangle and ADRs. To view this video click here: https://ecancer.org/journal/12/809-awareness-and-compliance-with-pharmacovigilance-requirements-amongst-UK-oncology-healthcare-professionals.php.

## Supplementary table

**Table 1.** Percentage of questions skipped.

Question	Percentage skipped (%)
1	0
2	0
3	2
4	4
5	7
6	10
7	10
8	10
9	11

## Appendix: List of questions

1. What is your Job Role in Oncology?
  Registrar
  Consultant
  Pharmacist
  Nurse
  Other

### Section 1: Adverse Events and Yellow cards

2. How many adverse events do you report per year if any?
  Never reported an AE (if selected the survey will skip to 5.)
  1
  2-4
  5-9
  10+
3. Do you report all serious adverse events for established medicines and vaccines?
  These would be considered to include reactions which are:
  − fatal
  − life-threatening
  − a congenital abnormality,
  − disabling or incapacitating
  − resulting in prolonged hospitalisation
    - Yes
    - No
4. What method do you use to report AEs to the MHRA? (Select all that apply)
  - Yellow Card App
  - Yellow card Post
  - Yellow card Website
  - MHRA Phone
  - Directly to Pharmaceutical company
  - In house/trust/hospital reporting scheme
  - Other
5. If you do not report all adverse events, what are your main reasons for this? (Select all that apply)
  - Did not know it was my responsibility
  - Process is too complex
  - Do not understand process
  - Follow up phone calls/emails/letters inconvenient
  - Time consuming
  - Did not think it was necessary/not serious enoughIt is difficult to decide when/what to report
  - Never had a patient with a suspected adverse event

### Section 2: Black Triangle

6. Which of the following options best describes the purpose of the black triangle?
  - I don’t know
  - Appears on European medication that is new to the market or when the safety profile is not fully established, indicating all adverse events should be reported.
  - Appears on European medication that is new to the market or when the safety profile is not fully established, indicating only serious or unexpected adverse events should be reported.
  - Appears on European medication which has an established safety profile.
7. After how long do you think the black triangle is removed?
  - 1 year
  - 3 years
  - 5 years
  - When the safety of the drug is established
8. Once a black triangle has been removed, which events should be reported to the MHRA? (Select all that apply)
  - All adverse events
  - Serious adverse events
  - Unexpected adverse events
  - Nothing
9. Does your adverse event reporting differ depending on whether the medicine has a black triangle or not?
  - Yes
  - No

### Section 3: Prescribing Information

10. Please select how often you use each source of information for prescribing (select more than one if applicable)
  **Table:**
  	Regularly used	Occasionally used	Rarely used	Never used
  Abbreviated PI attached to printed materials or journal adverts from Pharmaceutical companies				
  British National Formulary (BNF/BNFC)				
  Company website				
  Electronic Medicines Compendium (EMC) (SmPC)				
  Indication specific app				
  Local guidance				
  National guidance				
  Other				
11. For the sources that you use how do you access them (select more than one if applicable)
  **Table:**
  	Hard copy	App	Web browser	Don’t use
  Abbreviated PI attached to printed materials or journal adverts from Pharmaceutical companies				
  British National Formulary (BNF/BNFC)				
  Company website				
  EMC (SmPC)				
  Indication specific app				
  Local guidance				
  National guidance				
  Other				
12. When using printed materials from a company, do you check that you are using the most up-to-date abbreviated PI before prescribing?
  - Do not use this source
  - Yes
  - No
